# Comparison between modified lateral supraorbital approach and pterional approach in the surgical treatment of middle cerebral artery aneurysms

**DOI:** 10.1186/s41016-018-0110-2

**Published:** 2018-02-17

**Authors:** Zhouqing Chen, Xiaoou Sun, Tai Lu, Zhengyang Lu, Ming Jiang, Chongshun Zhao, Wanchun You, Yun Zhu, Zhong Wang

**Affiliations:** Department of Neurosurgery, The First Affiliated Hospital of Soochow University, 188 Shizi Street, Suzhou, Jiangsu Province 215006 China

**Keywords:** Aneurysm clipping, Middle cerebral artery aneurysms, Modified lateral supraorbital approach, Lateral supraorbital approach, Pterional approach

## Abstract

**Background:**

The Middle cerebral artery (MCA) aneurysm is a common type of craniocerebral aneurysm that is prone to rupture and high mortality. The classic surgical approaches are the Pterional approach and the Lateral Supraorbital (LSO) approach, but there are shortcomings.

**Methods:**

This study retrospectively analyzed clinical and imaging data from 181 patients with MCA aneurysm clipping in the Department of Neurosurgery, First Affiliated Hospital of Soochow University between 2011 and 2017. Statistical analysis using parametric and nonparametric tests showed that *P* values below 0.05 were considered statistically significant.

**Results:**

The preoperative GCS score (*P* = 0.003), Hunt-Hess scale (*P* < 0.001) and the operating habits of the surgeon (*P* < 0.001) affected the surgeon to choose a surgical approach. The choice of two surgical methods on the operation time (*P* < 0.001), skin incision (*P* < 0.001), complications (*P* = 0.026), tracheotomy (*P* = 0.014), prognosis (*P* = 0.002) were significantly different. Different surgical approaches (*P* = 0.002), Hunt-Hess scale (*P* < 0.001), GCS scale (*P* < 0.001), GCS sorse (*P* < 0.001), skin incision (*P* = 0.031) and complications (*P* < 0.001) are closely related to the prognosis of patients.

**Conclusions:**

Modified LSO approach provides another surgical approach for MCA aneurysm clipping, while avoiding the drawbacks of the LSO approach in the clipping of MCA distal aneurysm.

## Background

Middle Cerebral Artery (MCA) aneurysm is a common type of craniocerebral aneurysm, the incidence of which accounted for 20% of all aneurysms. Ruptured Middle Cerebral aneurysms have high morbidity and mortality [1]. Currently the main treatment of aneurysms includes clipping and coil interventional embolization. Here, we mainly explore the surgical approach to the middle cerebral aneurysm.

Yasargil introduced the Pterional or Frontal-temporo-sphenoidal approach since the 1970s, which is widely used in MCA aneurysm clipping [2]. Many cerebral aneurysms and saddle tumors were treated with the Lateral Supraorbital (LSO) approach when J. Hernesniemi and his colleagues innovated the use of the LSO approach [3]. In addition, the Pterional keyhole and Lateral supraorbital keyhole and other surgical approaches are also applicable to clinical [4–6]. These surgical methods will undoubtedly bring more possibilities for the director medical treatment of patients, but various surgical methods have their own advantages and disadvantages. The Pterional approach creates larger skin incisions in patients, at the same time, the atrophy of temporal muscle and the necrosis of bone flap affect the patient’s appearance and even lead to depression [7]. The advantage of the Pterional approach is that it that it allows more space around the aneurysm and is easy for beginners to master. The keyhole approach requires higher surgical skills and surgical equipment, and the smaller exposure to the aneurysm rupture could make surgery difficult. The advantage of the keyhole approach is that the skin incision is small and the effect on the appearance is slight as compared to the Pterional approach. The LSO approach also has a smaller surgical incision and is easy for the operator to master [3]. However, the traditional LSO approach has difficulties clipping the MCA aneurysm, especially in the treatment of the MCA distal aneurysm or posterior MCA aneurysm.

Here we describe the Modified Lateral Supraorbital approach for the treatment of MCA aneurysms. We analyzed the data to the modified lateral supraorbital approach and the Pterional approach in the MCA aneurysm surgical treatment. It is expected that this modified approach will result in a smaller surgical incision, a larger surgical space, and reducing the limitation on the MCA distal aneurysm or posterior MCA aneurysm.

## Methods

### Patient population

A retrospective study of 181 cases of MCA aneurysms, from January 2011 to May 2017, Department of Neurosurgery, First Affiliated Hospital of Soochow University. All patients via the modified LSO approach and some patients via the pterional approach were operated on by the senior author (Zhong Wang as surgeon physician A) (Table 1). The other patients were operated by other senior authors (Surgeon physician B) useing the Pterional approach (Table 1). The preoperative and postoperative radiological examination measurements and analyses were based on CTA and CT. Digital subtraction angiography detection was used if some initial diagnosis was uncertain or the operation required a detailed spatial position. Our hospital MCA aneurysm surgical approach only cover modified LSO approach or Pterional approach. The classic LSO approach used in other anterior circulation aneurysm or basilar artery aneurysm eliminated some cases of MCA aneurysms involving interventional therapy or abandonment of surgery.

**Table 1:** The relationship between two surgical approaches and some ralated factors

Varibles	Modified LSO group (No. of patients)	Pterional group (No. of patients)	*P* value
Sex			0.065
Female	21	91	
Male	6	63	
Age, ys			0.878
Range	39–73	28–81	
Mean ± SD	56.1 ± 10.1	56.4 ± 11.0	
Neck stiffness			0.196
Yes	18	82	
No	9	72	
Hunt-Hess, n (%)			0.004
Good grade (0–2)	26	108	
Poor grade (3–5)	1	46	
GCS			0.003
≤ 12	1	48	
>12	26	106	
Surgeon physician			0.001
A	26	21	
B	1	133	
Operation time (mean minutes)			0.001
Mean ± SD	146 ± 36.7	240 ± 80.8	
Skin incision (Average length cm)			0.001
Mean ± SD	9.81 ± 2.6	15.68 ± 3.7	
Complication			0.026
Yes	2	42	
No	25	112	
Tracheotomy			0.014
Yes	0	29	
No	27	125	
Prognosis			0.002
Good	25	97	
Bad	2	57	

Twenty-seven patients (14.9%), 21 females and 6 males, were treated via the modified LSO approach. One hundred and fifty-four patients (85.1%), 91 females and 63 males, were treated via the Pterional approach. The mean age was 56.1 ± 10.1 years and ranged from 39 to 73 years in modified LSO group. The mean age was 56.4 ± 11.0 years and ranged from 28 to 81 years in Pterional group. Hunt-Hess (HH) scale was categorized as good grade (HH 0–2), or poor grade (HH 3–5) [8]. Neck stiffness is considered to be the standard of assessment of meningeal irritation signs.

Prognosis after surgery was evaluated using the Glasgow Outcome scale (GOS) during follow-up. The score applies to patients with brain injury and allows an objective assessment of their recovery. The specific content of this score is: GOS = 1, Death; GOS = 2, Persistent vegetative state; GOS = 3, Severe disability; GOS = 4, Moderate disability; GOS = 5, Low disability. The prognosis was evaluated as favorable (GOS = 4–5), unfavorable (GOS = 2–3), or dead (GOS = 1) [9]. In addition, we defined favorable as a good prognosis, unfavorable and dead as a bad prognosis.

### Bone window

**Fig. 1:**
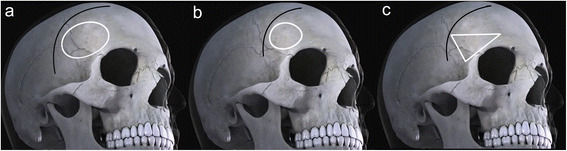
Contrast Pterional approach, LSO approach and modified LSO approach bone window shape in craniotomy. **a** represents a conventional Pterional approach, **b** represents a classical LSO approach, and **c** represents a modified LSO approach

We draw three kinds of approach bone window: Pterional approach, classical LSO approach and Modified LSO approach through photoshop software (Fig. 1). Obviously, the Pterional approach skin incision the longest, followed by modified LSO approach, the shortest of classical LSO approach. For the conversion of the area size, the Pterional approach and the classical LSO approach use the elliptical area formula (*S = πab*), while the modified LSO approach using the triangular area formula (S = 1/2ab). Based on past experience, we assume that the bone flap of the Pterional approach is similar the oval, the long axis is 5 cm, the short axis is 4 cm and the area is 15.7cm^2^. We assume that the bone flap of the classical LSO approach is similar the oval, the long axis is 4 cm, the short axis is 3 cm and the area is 9.42cm^2^. We assume that the bone flap of the modified LSO approach is right-angled triangles, the long right-angled edges is 4 cm, the short right-angled edges is 3 cm and the area is 6cm^2^.

After comparing the three areas, it is clear that bone flap of the Pterional approach is the largest and the bone flap of modified LSO approach is the minimum. The above analysis we can get this understanding, modified LSO approach has a shorter skin incision and smaller bone window. No matter how we deal with MCA aneurysms, we have no choice of the classical LSO approach. Because the classic LSO approach bone window is not conducive to solve problem that the MCA aneurysm position is later than other anterior circulation aneurysm. In order to get more operating space, we have to choose to the modified LSO approach.

**Fig. 2:**
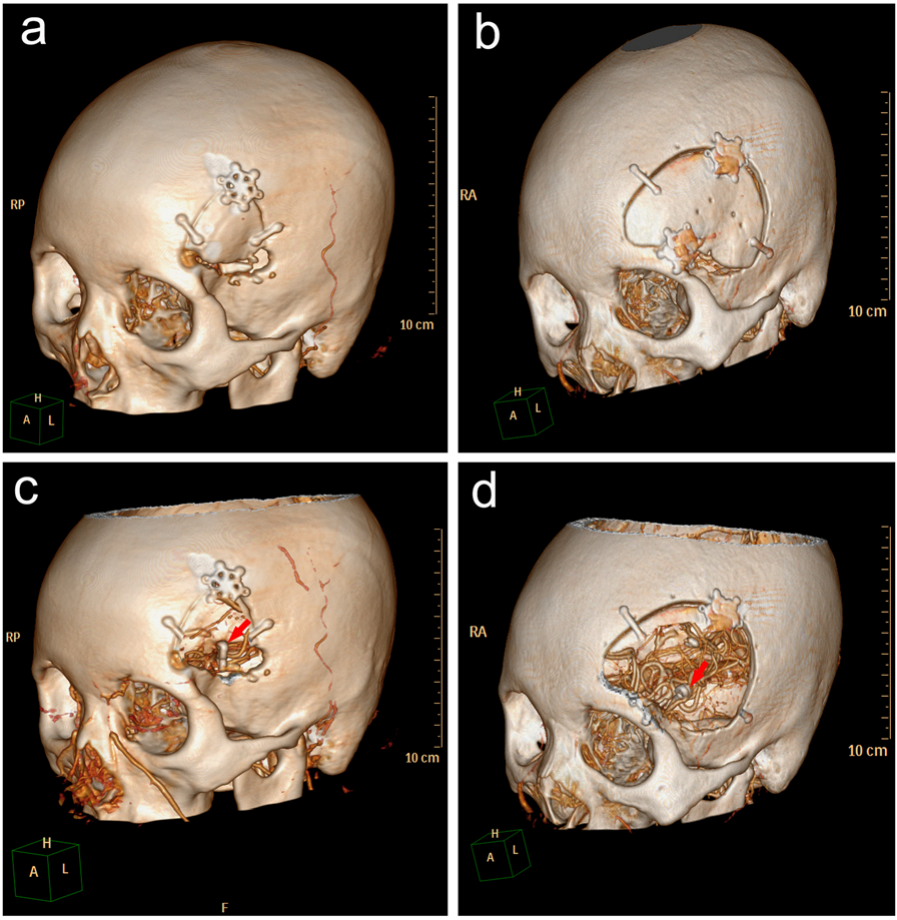
Contrast modified LSO approach and Pterional approach bone window shape in middle aneurysm clipping surgery. **a** and **b** represent the shape of the bone flap of the modified LSO approach and the Pterional approach, respectively. **c** and **d** are **a** and **b** through the software to remove the bone flap obtained from the perspective of the picture. Aneurysm clips are marked with red arrows

The skull bone window of two different patients was observed by 3D–CTA synthetic imaging technique: Modified LSO approach and Pterional approach (Fig. 2). All bone window images were obtained from patients with MCA aneurysm after a month of surgery to review CTA. Fig. 2a and b represent the shape of the bone flap of the modified LSO approach and the Pterional approach, respectively. The bone flap in Fig. 2a and b was removed by software to obtain Fig. 2c and d. Through the bone window can be seen MCA aneurysm was clamped and aneurysm clip, in Fig. 2c and d. Get different bone window data through 3D–CTA software. Figure 2a is an equilateral triangle with a side length of 4 cm and an area of 6.92 cm. Figure 2b is similar the oval, the long axis is 6.5 cm, the short axis 5.5 cm, the area is 28.06 mm.

### Preoperative planning

Surgical planning was based primarily on preoperative 3D–CTA, when the initial diagnosis was ambiguous or the aneurysm was complex, then further DSA was examined. Factors affecting the choice of surgical approach are the location and morphology of aneurysms; the degree of coma in patients, intracranial pressure and surgical practices of the surgeon, the hairline level surgical incision size, and the general surgical incision in the hairline, there is a better cosmetic effect in the postoperative. Preoperative cerebral hemorrhage more, coma deep, high intracranial pressure were preferred Pterional approach, large bone flap decompression effect.

### Surgical technique

In the treatment of MCA aneurysms, we mainly take two surgical approaches: modified LSO approach and Pterional approach. Pterional approach is widely known, and we focus on modified LSO approach.

**Fig. 3:**
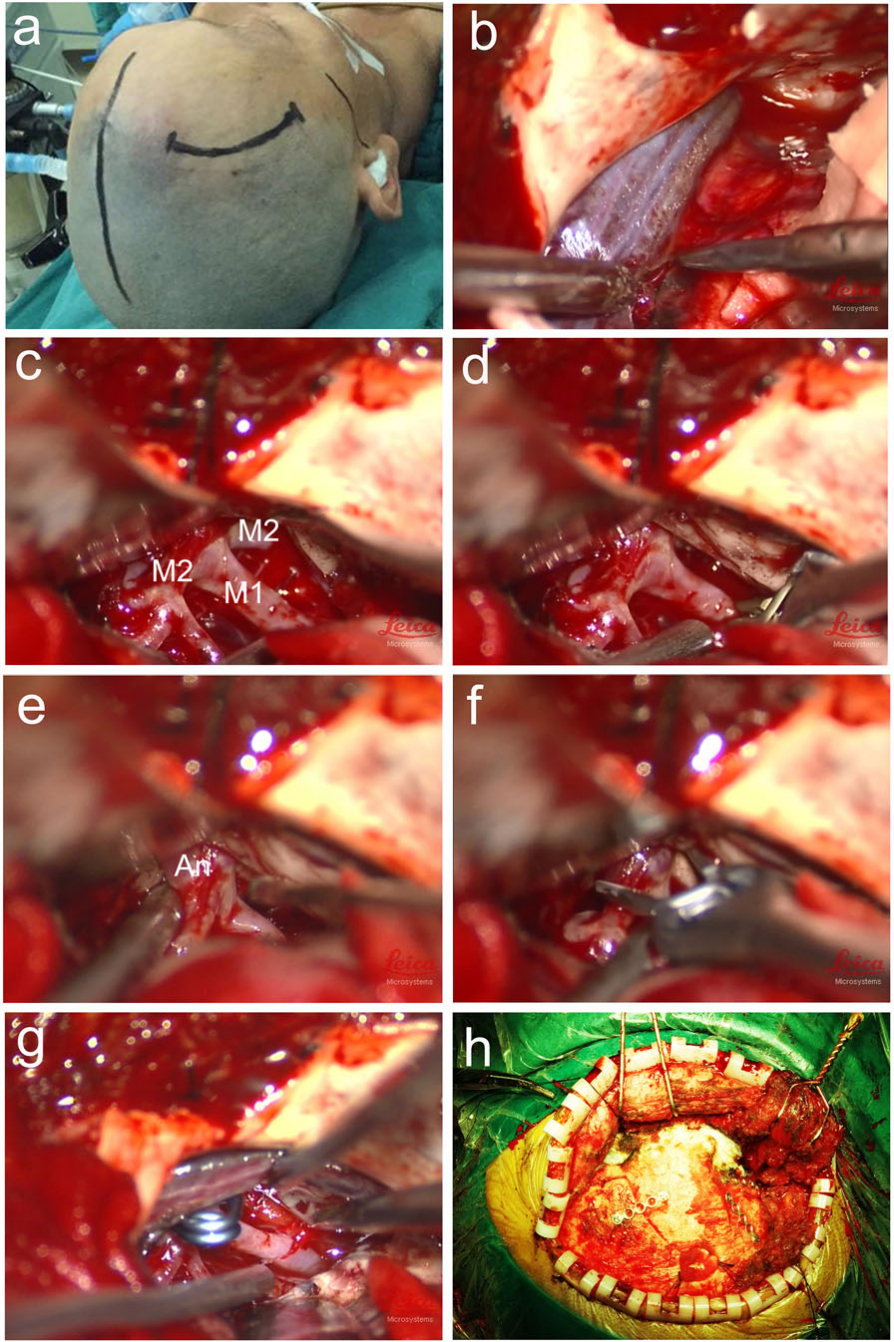
Operation flow chart of MCA aneurysm clipping by modified LSO approach. **a** is the skin incision pattern. **b** and **c** represent the presentation of MCA aneurysms. **d** represents temporary blocking aneurysm proximal blood vessels. **e-g** represents the MCA aneurysm clipping process. **h** represents bone flap reduction

The patient is supine on the operating table, with a pillow pad on the craniotomy side of the shoulder. According to the preoperative 3D osteotomy program to determine the best head rotation angle, the general head rotation angle of 15–45 degrees, the head rotation to the opposite side, the vertex slightly downward tilt, neck extension. If it is possible, skin incision left behind the hairline. Skin incision is very shortly, down will not exceed the level of zygomatic arch (Fig. 3a). Cut the skin, the skin from the bone flap, to avoid injury to the facial nerve branch, the front of the temporal muscle to the bottom of the downward, until the exposed edge of the eyebrow outside. In the skull drill three burr holes, eyebrows outside the edge to ensure that there is a burr hole, burr hole spacing equal to the bone flap was equilateral triangle or right angle triangle. The length of the bone flap is generally 4 × 4 × 4 cm or 3 × 4 × 5 cm. In order to better reveal the lateral fissure, if necessary, bite part of the lateral sphenoid ridge. Dura mater suspended in the bone flap or cap-shaped aponeurosis, dural opening turned to the bottom. If the intracranial pressure is too high, we first release cerebrospinal fluid of chiasmatic cistern and suprasellar cistern, or even open the lamina terminalis to further reduce intracranial pressure, to facilitate better surgical operation, for reducing the brain tissue traction. Sharp knife sharp separation and bipolar coagulation bluntly separation, open the side crack, revealing MCA aneurysm (Fig. 3b,c). Temporary blocking aneurysm proximal blood vessels, to prevent MCA aneurysm rupture of bleeding during surgery (Fig. 3d). Carefully separate the aneurysm around the arachnoid adhesions, reveal the aneurysm neck, selecting the appropriate aneurysm clip and the appropriate angle to clamp the aneurysm (Fig. 3e-g). Bone flap reduction, sutured temporal muscle and skin incision (Fig. 3h).

### Statistical analyses

Statistical analyses were performed using SPSS software version 21.0. The normality of quantitative data distribution was tested using the Kolmogorov-Smirnov test. Categorical variables are presented as percentages or medians and continuous variables are presented as means. The analysis of categorical variables was tested using the x-square test and the analysis of continuous variables was tested using the ANOVA test (parametric data) and Mann-Whitney U test (nonparametric data). A *p* value<0.05 was considered significant difference.

## Results

### The choice and effects of surgical approaches

All the MCA aneurysm patients were preoperative data were analyzed to obtain the following results. There was no significant difference in the choice of sex (*P* = 0.065), age (*P* = 0.878) and neck stiffness (*P* = 0.196) between the two surgical approaches (Table 1). The choice of surgical approaches was mainly due to Hunt-Hess scale (*P* = 0.004), GCS score (*P* = 0.003) and surgeon physician (*P* < 0.001), with significant differences. Retrospective study of two kinds of surgical approach in the time, skin incision, complications, tracheotomy and prognosis and so on. There were significant statistical differences between the two surgical approaches on the operation time (*P* < 0.001), skin incision (*P* < 0.001), postoperative complications (*P* = 0.026), tracheotomy (*P* = 0.014) and prognosis (*P* = 0.002) (Table 1).

### Surgical outcomes

In order to observe the prognosis of patients, the average follow-up was 3.2 months (0–24 months). Of the 181 patients selected, only one was an unruptured aneurysm and the remaining 180 were ruptured aneurysms. A favorable outcome was defined as good and fair clinical outcome (GOS 5 and 4), an unfavorable outcome was defined as bad and unfair clinical outcome (GOS 3 and 2). There were 122 patients with favorable outcome (67.4%), 48 patients with unfavorable outcome (26.5%), and 11 patients died (6.1%). Some factors, such as sex (*P* = 0.732), age (*P* = 0.066), neck stiffness (*P* = 0.271), hypertension (*P* = 0.716) and operation time (*P* = 0.111) had no statistically significant difference in MCA aneurysm surgery (Table 2). However, these factors such as surgical approach selection (*P* = 0.002), Hunt-Hess scale (*P* < 0.001), GCS scale (*P* < 0.001), skin incision length (*P* = 0.031) and complications (*P* < 0.001), the outcome of patients with significant differences (Table 2).

**Table 2:** Effects of different influencing factors on prognosis of patients

Varibles	Favorable	Unfavorable	Dead	*P* Value
Approach, n (%)				0.002
LSO	25 (92.6)	2 (7.4)	0 (0)	
Pterional group	97 (63.0)	46 (29.9)	11 (7.1)	
Sex, n (%)				0.732
Male	46 (66.7)	17 (24.6)	6 (8.7)	
Female	76 (67.9)	31 (27.7)	5 (4.5)	
Age, y				0.066
<50	41 (77.4)	10 (18.9)	2 (3.8)	
≥ 50	81 (63.3)	38 (29.7)	9 (7.0)	
Hunt-Hess, n (%)				0.001
Good grade (0–2)	100 (74.6)	30 (22.4)	4 (3.0)	
Poor grade (3–5)	22 (46.8)	18 (38.3)	7 (14.9)	
GCS				0.001
≤ 12	23 (46.9)	19 (38.8)	7 (14.3)	
>12	99 (75.0)	29 (22.0)	4 (3.0)	
Neck tough				0.271
Yes	65 (64.4)	28 (27.7)	8 (7.9)	
No	57 (71.3)	20 (25.0)	3 (3.8)	
Hypertension				0.716
Yes	66 (68.8)	24 (25.0)	6 (6.3)	
No	56 (65.9)	24 (28.2)	5 (5.9)	
Skin incision (cm)				0.031
<15	42	12	0	
≥ 15	80	36	11	
Operation time (mins)				0.111
<180	34	8	2	
≥ 180	88	40	9	
Complication				0.001
No	119	13	5	
Hematoma	0	12	4	
Infection	1	8	1	
Infarction	1	10	0	
Others	2	5	1	

## Discussion

Pterional approach has been used in clinical for many years as the gold standard in the treatment of vascular diseases and tumors located in the saddle nodules, supra- and sellar area, Sylvian fissure, the superior part of the basilar artery and clivus, etc. In addition, a large number of new surgical approaches have been used in clinical practice. At present, the mainstream of surgical approach in the clipping of MCA aneurysm include: LSO approach, Pterional keyhole approach, lateral supraorbital keyhole approach, etc. [6, 7, 10]. The classic Pterional approach has a large bone window conducive to reveal the surgical field of vision and reduce the risk of surgery. However, the larger surgical skin and bone flap exposure also brings unfavorable factors such as skin beauty problems, temporal muscle atrophy, longer operation time and prognosis wound recovery time. Many senior experts have made improvements in surgical methods to make surgical trauma smaller and at the same time can’t bring more technical problems to surgeons. J. Hernesniemi from Helsinki University Central Hospital used LSO approach for the first time and promoted the surgical procedure [3]. This surgical approach reduces the length of the skin incision and bone areas, greatly reducing the operation time, improve the patient’s postoperative aesthetic and temporal muscle atrophy. In addition, some keyhole approach also has a smaller trauma, but the surgical technique is more complex. Different surgical approaches have their own advantages and disadvantages depending on the specific circumstances. For the surgeon, the most familiar surgical approach may be the best surgical approach [3, 11].

In the case of MCA aneurysm surgery, the Pterional approach is still feasible, but the LSO approach seems to be more difficult to reach the MCA distal aneurysm, such as M2 segment aneurysm or even further. In order to solve this problem, we will be on the LSO approach temporal skin incision near the ear, while taking equilateral triangular or right triangular bone flap, access to more on the brain side of the operating space. With such improvements, it is surprisingly found that this improvement is feasible.

The modified LSO approach did not significantly increase the length of the skin incision. At the same time, the temporal muscle was separated from the bone, leave a small piece of temporal muscle at the insertions of temporal muscle so as to facilitate the temporal muscle suture reduction. For the bone window area, the same length and height of the triangular bone area was significantly smaller than the same long axis and short axis of the oval area. We confirmed that the triangular bone window had a smaller area of bone flap during postoperative 3D–CTA bone window reconstruction (Fig. 2). We retrospectively studied the improvement of the modified LSO approach and Pterional approach in the data, we found that improved approach could shorten the operation time, reduce skin incision, reduce bone window area. The modified LSO approach technically has no difficulty in operation, the operator is easy to grasp. When faced with a posterior MCA aneurysm or a complex large MCA aneurysm, we use a right-angled triangular bone flap, and one of the burr holes is shifted backwards to allow the sylvian fissure to obtain more operating space.

However, the modified LSO approach also has some limitations, such as preoperative patients with deep coma, high intracranial pressure patients and patients with scheduled bypass surgery should not use the surgical approach. Therefore, the severity of subarachnoid hemorrhage in patients, the choice of two groups of patients there is a certain degree of bias. Our hospital MCA aneurysm surgical approach only modified LSO approach or Pterional approach. The modified LSO approach and classical LSO approach can’t be retrospectively controlled study. There is a certain degree of difference in the retrospective study and analysis, the collection of different medical records and the lack of uniform standards. At the same time, different surgeons have different surgical levels. Although we use the authority of the software to analyze the available clinical data, but the data collection is still insufficient.

## Conclusion

The modified LSO approach may be more suitable for MCA aneurysm surgery than the classic LSO approach, especially in the MCA distal aneurysm. In addition, modified LSO approach reduces MCA aneurysm surgery time, skin incision length, compared with Pterional approach. This approach solves the difficulties of the classical LSO approach in handling MCA distal aneurysms and complex MCA aneurysms, while avoiding greater surgical trauma of the Pterional approach. Therefore, we believe that a modified LSO approach provides a new surgical approach for MCA aneurysms.

## Abbreviations

CT: Computed tomography
CTA: Computed tomography angiography
DSA: Digital subtraction angiography
HH: Hunt-Hess
LSO: Lateral supraorbital
MCA: Middle cerebral artery

## Authors’ information

Department of Neurosurgery, The First Affiliated Hospital of Soochow University.
